# VAChT overexpression increases acetylcholine at the synaptic cleft and accelerates aging of neuromuscular junctions

**DOI:** 10.1186/s13395-016-0105-7

**Published:** 2016-10-05

**Authors:** Satoshi Sugita, Leland L. Fleming, Caleb Wood, Sydney K. Vaughan, Matheus P. S. M. Gomes, Wallace Camargo, Ligia A. Naves, Vania F. Prado, Marco A. M. Prado, Cristina Guatimosim, Gregorio Valdez

**Affiliations:** 1 Virginia Tech Carilion Research Institute, Virginia Tech, Roanoke, VA USA; 2 Department of Biological Sciences, Virginia Tech, Blacksburg, VA USA; 3 Graduate Program in Translational Biology, Medicine, and Health, Virginia Tech, Blacksburg, VA USA; 4 Virginia Tech Postbaccalaureate Research and Education (VT PREP) Scholar, Virginia Tech, Blacksburg, VA USA; 5 Departamento de Morfologia, Instiuto Ciencias Biologicas, Universidade Federal de Minas Gerais, Belo Horizonte, Minas Gerais Brazil; 6 Departamento de Fisiologia e Biofísica, Instiuto Ciencias Biologicas, Universidade Federal de Minas Gerais, Belo Horizonte, Minas Gerais Brazil; 7 Robarts Research Institute, Department of Physiology and Pharmacology, Department of Anatomy & Cell Biology, Schulich School of Medicine & Dentistry, University of Western Ontario, London, ON N6A5K8 Canada

**Keywords:** Neuromuscular junction, Acetylcholine, VAChT, Aging, ALS, Synapse, Cholinergic transmission, Motor neuron

## Abstract

**Background:**

Cholinergic dysfunction occurs during aging and in a variety of diseases, including amyotrophic lateral sclerosis (ALS). However, it remains unknown whether changes in cholinergic transmission contributes to age- and disease-related degeneration of the motor system. Here we investigated the effect of moderately increasing levels of synaptic acetylcholine (ACh) on the neuromuscular junction (NMJ), muscle fibers, and motor neurons during development and aging and in a mouse model for amyotrophic lateral sclerosis (ALS).

**Methods:**

Chat-ChR2-EYFP (VAChT^Hyp^) mice containing multiple copies of the vesicular acetylcholine transporter (VAChT), mutant superoxide dismutase 1 (SOD1^G93A^), and Chat-IRES-Cre and tdTomato transgenic mice were used in this study. NMJs, muscle fibers, and α-motor neurons’ somata and their axons were examined using a light microscope. Transcripts for select genes in muscles and spinal cords were assessed using real-time quantitative PCR. Motor function tests were carried out using an inverted wire mesh and a rotarod. Electrophysiological recordings were collected to examine miniature endplate potentials (MEPP) in muscles.

**Results:**

We show that VAChT is elevated in the spinal cord and at NMJs of VAChT^Hyp^ mice. We also show that the amplitude of MEPPs is significantly higher in VAChT^Hyp^ muscles, indicating that more ACh is loaded into synaptic vesicles and released into the synaptic cleft at NMJs of VAChT^Hyp^ mice compared to control mice. While the development of NMJs was not affected in VAChT^Hyp^ mice, NMJs prematurely acquired age-related structural alterations in adult VAChT^Hyp^ mice. These structural changes at NMJs were accompanied by motor deficits in VAChT^Hyp^ mice. However, cellular features of muscle fibers and levels of molecules with critical functions at the NMJ and in muscle fibers were largely unchanged in VAChT^Hyp^ mice. In the SOD1^G93A^ mouse model for ALS, increasing synaptic ACh accelerated degeneration of NMJs caused motor deficits and resulted in premature death specifically in male mice.

**Conclusions:**

The data presented in this manuscript demonstrate that increasing levels of ACh at the synaptic cleft promote degeneration of adult NMJs, contributing to age- and disease-related motor deficits. We thus propose that maintaining normal cholinergic signaling in muscles will slow degeneration of NMJs and attenuate loss of motor function caused by aging and neuromuscular diseases.

## Background

The organization and stability of synapses is dictated by the actions of pre- and postsynaptic organizing molecules, including neurotransmitters [1–3]. The vertebrate neuromuscular junction (NMJ), the synapse formed between α-motor neurons and skeletal muscle fibers, is under the influence of the neurotransmitter acetylcholine (ACh) [1]. In addition to promoting muscle contraction, several lines of evidence indicate that ACh acts as an anti-synaptogenic factor [4, 5]. ACh promotes dispersion of nicotinic acetylcholine receptors (AChR) from postsynaptic sites by inducing posttranslational changes and accelerating the endocytosis of AChRs [6]. In mice lacking ACh, developing muscles contain larger and more complex postsynaptic sites that are innervated by silent motor axons. These postsynaptic sites initially form but fail to mature in the absence of neural-derived agrin (z-agrin), a molecule with critical roles in stabilizing AChRs [7, 8]. These findings have led to the hypothesis that ACh acts in concert with z-agrin in sculpting and stabilizing postsynaptic sites [7].

The recent generation of transgenic mice with altered expression of the vesicular acetylcholine transporter (VAChT) has made it possible to examine the impact of varying levels of ACh throughout the lifespan of mice [9, 10]. VAChT functions to load ACh into synaptic vesicles, thereby regulating the amount of neurotransmitter released [11]. Young adult mice with reduced expression of VAChT (VAChT^KD^) have reduced cholinergic neurotransmission [10] while mice overexpressing VAChT (*ChAT–ChR2–EYFP*; herein called VAChT^Hyp^) [12, 13] release more ACh at cholinergic synapses and thus have heightened cholinergic transmission [9]. Both transgenic lines exhibit cognitive deficits, indicating that cholinergic circuits in the brain are highly sensitive to both increasing and decreasing synaptic ACh. In the lower motor system, decreasing synaptic ACh by approximately 70 %, as is the case in VAChT^KD^ mice, results in symptoms resembling myasthenia gravis [10]. These mice also exhibit moderate changes in the shape and distribution of synaptic vesicles in α-motor axon nerve endings [14]. Conversely, young adult VAChT^Hyp^ mice have enhanced motor endurance consistent with increased cholinergic tone [9].

During aging and throughout the progression of amyotrophic lateral sclerosis (ALS), neuromuscular function decreases suggesting that increasing the release of ACh may potentially preserve muscle tone [1, 15]. Here we used VAChT^Hyp^ mice to determine the contribution of chronically increasing synaptic ACh levels on developing, aging, and ALS-afflicted NMJs. We discovered that NMJs prematurely acquire age-related structural alterations in VAChT^Hyp^ mice. These structural changes at NMJs are accompanied by a moderate reduction in the size of muscle fibers and motor deficits in aged VAChT^Hyp^ mice. To determine if increased cholinergic transmission affects the progression of ALS-like pathology, we examined SOD1^G93A^ mice overexpressing VAChT. Increasing cholinergic transmission selectively affected male SOD1^G93A^ mice, prematurely causing motor deficits, degeneration of NMJs, and accelerating death of these mice. Together, these findings demonstrate that increasing cholinergic transmission accelerates the degeneration of NMJs during aging and progression of ALS-like pathology in a mouse model of the disease.

## Methods

### Mice

Several transgenic lines were used in these experiments. We obtained the following lines from The Jackson Laboratory: VAChT^Hyp^ (B6.Cg-Tg(Chat-COP4*H134R/EYFP,Slc18a3)6Gfng/J) [13], SOD1^G93A^ (B6.Cg-Tg(SOD1*G93A)1Gur/J) [16], ChAT-IRES-Cre (B6;129S6-Chat < tm1(cre)Lowl>/J) [17], tdTomato (B6;129S6-*Gt(ROSA)26Sor* ^*tm14(CAG-tdTomato)Hze*^/J) [18]. Mice overexpressing VAChT [13] contained several bacterial artificial chromosomes (BAC) modified to express channelrhodopsin from the ChAT locus. However, the BAC construct used to generate these transgenic mice also contained an intact VAChT locus, thus introducing additional functional copies of the VAChT gene. All transgenic mice overexpressing VAChT (VAChT^Hyp^) [13] were maintained as heterozygous and allowed free access to food and water. For histological and biochemical analysis of NMJs, muscle fibers, motor neurons, and motor axons, we used at least four mice per genotype per experiment. All experiments were carried out under NIH guidelines and animal protocols approved by the Virginia Tech Institutional Animal Care and Use Committee.

### Electrophysiological recordings

We recorded miniature endplate potentials (MEPPs) using an Axoclamp 2A amplifier. Recordings were band-passed filtered at 0.1 to 5 kHz, further amplified 50 times by a Cyberamp, and then sampled on a computer at a frequency of 100 kHz. Microelectrodes were fabricated from borosilicate glass using a Narishige puller (PN-30) and had resistances of 8–15 Mohms when filled with 3 M KCl. The microelectrodes were inserted into the muscle fiber in the endplate region to record MEPPs. Tetrodotoxin (100 nM) was added to the bath solution to prevent muscle contraction. The signals were digitized by a board from National Instruments (NIDAQ-MX) and acquired by the program WinEDR (John Dempster, University of Strathclyde). MEPP amplitudes were corrected to a standard resting potential of −70 mV. Approximately 100 MEPPs in five different synapses per muscle were analyzed. We used three muscles from three different animals per genotype at 4 months of age.

### Immunohistochemistry and histological analysis under confocal microscopy

#### Imaging NMJs

Mice were anesthetized with isoflurane and perfused transcardially with 4 % paraformaldehyde in phosphate buffered saline (PBS). The extensor digitorum longus (EDL) muscle was then dissected. Whole muscles were blocked for 1 h at room temperature (blocking solution; 1 % Triton X-100, 3 % BSA, 5 % goat serum in PBS) and then incubated with primary antibodies for 24 h in blocking solution to visualize AChRs, axons, and synaptic vesicles. Following staining with primary antibodies, muscles were washed three times with PBS-T (0.1 % Triton X-100) and incubated for 2 h with Alexa 488 or 555 conjugated α-bungarotoxin (fBTX, Life Technologies; 1:1000) and secondary antibodies. After washing with PBS-T, the muscles were whole-mounted onto slides using Vectashield (Vector Labs). The primary antibodies used were synaptotagmin-2 (znp-1, Zebrafish International Resource Center; 1:100) and VAChT (Millipore; 1:250). The secondary antibodies used were Alexa-647 anti-mouse IgG2a (Life Technologies; 1:1000) and Alexa-647 anti-guinea pig IgG (Life Technologies; 1:1000).

#### NMJ analysis

To analyze structural features at NMJs, maximum intensity projections of confocal stacks were created using ZEN software (Zeiss). We analyzed structural features following the criteria previously described by Valdez et al. [19]. Briefly, fragmented AChRs are defined as five or more AChR clusters in small islands with round shape and/or a segment of the postsynapse. It also includes NMJs with small and/or irregularly shaped AChR clusters. Full or partial denervation describes postsynaptic sites not appropriately opposed by the nerve terminal. Multiple innervations are the simultaneous innervation of the postsynapse by two or more axons. A nerve sprout occurs when the nerve extends beyond AChR clusters in any direction. Colocalization is the extent of pre- and postsynaptic apposition measured using ZEN software. To quantify the size of NMJs, the region occupied by AChRs was measured using ImageJ software.

#### Fluorescence intensity analysis

To analyze the fluorescence intensity of VAChT immunostaining at NMJs, maximum intensity projections of confocal stacks were created using ZEN software (Zeiss). Using Zen Black software (Zeiss), individual NMJs were outlined and the mean fluorescence intensity was determined by Zen Black software with background fluorescence subtracted. The mean fluorescence intensity for individual NMJs was averaged to find the overall fluorescence intensity for each animal.

### Muscle fiber diameter/central nuclei

The tibialis anterior (TA) muscle was dissected from perfused mice, transferred into a 30 % sucrose solution for 2 days, and cut using a cryostat at 14-μm thickness. To visualize muscle fiber size and location of nuclei, the sections were stained by first blocking for 1 h at room temperature (0.1 % Triton X-100, 3 % BSA, 5 % goat serum in PBS) and then incubated with an antibody against laminin (L9393, Sigma; 1:100) for 24 h in blocking solution. The sections were washed three times in PBS and incubated for 3 h with secondary antibodies (Alexa-568 anti-rabbit IgG, Life Technologies; 1:1000). After washing with PBS, sections were incubated with 4′,6-diamidino-2-phenylindole (DAPI; Sigma; 1:1000), washed with PBS, and mounted using Vectashield. The area outlined by laminin was measured using ImageJ software. At least 300 muscle fibers per mouse were randomly selected and used for this analysis. Myonuclei located in the center of muscle fibers were counted. At least 1000 nuclei per mouse were counted.

### Nerve counts

To visualize motor axons, Chat-Cre;tdTom mice were used. The peroneal nerve was dissected from perfused mice and embedded in a tissue-freezing medium (Tissue-Tek). The specimens were cut in a cryostat at 10-μm thickness. Sections were blocked for 1 h at room temperature (0.1 % Triton X-100, 3 % BSA, 5 % goat serum in PBS) and subsequently incubated with antibodies for both neurofilament (smi-312, Covance; 1:1000) and S-100 (Z0311, Dako; 1:400) for 24 h in blocking solution. Sections were then washed three times with PBS for 5 min each and incubated with secondary antibodies (Alexa-488 anti-mouse IgG1, Alexa-647 anti-rabbit IgG, Life Technologies; 1:1000). After washing with PBS, sections were mounted in Vectashield. Axons expressing tdTom and neurofilament were counted as motor axons. Axons labeled with only neurofilament are sensory axons.

### Expression analysis using quantitative PCR

Mice were first anesthetized with isoflurane, and then the muscles and spinal cord regions, including cervical, thoracic, lumbar, and sacral regions, were dissected and flash frozen in liquid nitrogen. Total RNA was prepared using Aurum Total RNA Mini kit (Bio-Rad) following the manufacturer’s instructions. Complementary DNA (cDNA) was synthesized from 500 ng of total RNA using the iScript cDNA synthesis kit (Bio-Rad). PCR amplification was performed on the Bio-Rad CFX Connect Real-Time System (Bio-Rad) using iTaq Universal SYBR Green Supermix (Bio-Rad). All primers used in this study are listed in Table 1.

**Table 1:** PCR primers

Gene	Fw (5′–3′)	Rv (5′–3′)
GAPDH	CCCACTCTTCCACCTTCGATG	GTCCACCACCCTGTTGCTGTAG
AChE	CTACACCACGGAGGAGAGGA	CTGGTTCTTCCAGTGCACCA
AChRγ	GCTCAGCTGCAAGTTGATCTC	CCTCCTGCTCCATCTCTGTC
AChRε	GCTGTGTGGATGCTGTGAAC	GCTGCCCAAAAACAGACATT
MuSK	CCACACTGCGTGGAATGAGC	CTCTGCAAATGGGCATGGG
LRP4	GGCAAAAAGCAGGAACTTGT	TCTACCCAGTGGCCAGAACT
Rapsyn	GTGCCATGGAGTGTTGTGAG	CGGTTTCCGATCTCAGTCAT
Dok7	GGGTACTGGGCTGGAGTCTT	TCGGACGATGCAGTCAAACA
CDK5	GCCAGACTATAAGCCCTACCC	GTCAGAGAAGTAGGGGTGCT
MyHC2A	GAGTGAGCAGAAGCGGAATGCT	GCGGAACTTGGATAGATTTGTG
MyHC2B	CACCTGGACGATGCTCTCAGA	GCTCTTGCTCGGCCACTCT
MyHC2X	GCTAGTAACATGGAGGTCA	TAAGGCACTCTTGGCCTTTATC
Myogenin	GCACTGGAGTTCGGTCCCA	GTGATGCTGTCCACGATGGA
Foxo1	GAGTTAGTGAGCAGGCTACATTT	TTGGACTGCTCCTCAGTTCC
Atrogin-1	GCAGCAGCTGAATAGCATCCA	GGTGATCGTGAGGCCTTTGAA
Agrin-8	CTTTGATGGGCGGACCTACA	CGCTTTCTCAGCTGGGATCT
Agrin-11	CAGTGGGGGACCTAGAAACAC	TTTCAGGGCTCTCAGTCACAG
Agrin-19	CTTTGATGGGCGGACCTACA	AGTTTCAGGGGCTGGGATCT
Chat	CCTGGATGGTCCAGGCACT	GTCATACCAACGATTCGCTCC
VAChT	GAGAGTACTTTGCCTGGGAGGA	GGCCACAGTAAGACCTCCCTTG

### SOD1^G93A^ copy number analysis

DNA isolation was performed with the DNeasy Blood and tissue kit (Qiagen). Changes in transgene copy numbers were evaluated using TaqMan probe-based quantitative real-time PCR by determining the difference in threshold cycle (ΔCT) between the transgene (hSOD1) and an internal positive control gene. Primers for hSOD1 were as follows: IMR9665 or Forward = 5′-GGGAAGCTGTTGTCCCAAG-3′; and IMR9666 or Reverse = 5′-CAAGGGGAGGTAAAAGAGAGC-3′. The TaqMan probe used for hSOD1 is 13854 = 5′-CTGCATCTGGTTCTTGCAAAACACCA-3′. The primers for the internal positive control gene were as follows: IMR1544 or Forward = 5′-CACGTGGGCTCCAGCATT-3′; and IMR3580 or Reverse = 5′-TCACCAGTCATTTCTGCCTTTG-3′. The TaqMan probe for the internal positive control gene is TmoIMR0105 = 5′-CCAATGGTCGGGCACTGCTCAA-3′. These primers and probes are described in the Jackson Laboratories protocol for genotyping hSOD1 transgenic mice. We made minor modifications to the protocol to compare levels of hSOD1 between mice. We used 5 ng of genomic DNA, the TaqMan Universal PCR Mastermix (Thermo Fisher Scientific), and the Bio-Rad CFX Connect Real-Time System (Bio-Rad) to amplify hSOD1. The following PCR settings were used: 50 °C for 2 min, 95 °C for 10 min, 40 PCR cycles of 95 °C for 15 s, 60 °C for 1 min. To determine relative copy numbers, we compared the ΔCT value between SOD1^G93A^ and SOD1^G93A^;VAChT^Hyp^ for hSOD1 after subtracting the CT value of the internal control gene for each sample. The same ΔCT value indicates that there is no difference in the number of hSOD1 between SOD1^G93A^ and SOD1^G93A^;VAChT^Hyp^ mice.

### Behavioral tests

#### Hanging test

We examined motor function using an inverted grid hanging test [20]. The mice were placed on the center of a wire grid, which was mounted 25 cm above the table. After gently inverting the wire grid, we recorded the time the mouse remained hanging from the wire mesh. Each mouse was tested three times with at least 5-min breaks between trials. When comparing the ability of mice to stay on the wire mesh, we only used the maximum time they spent hanging for each trial.

#### Rotarod test

Mice were placed on a rotarod (Ugo Basile Instruments). The time that the mice were able to spend on the rotating platform was recorded. The following settings were used: for 400-day-old VAChT^Hyp^ and wild-type mice, acceleration was set to 8.0 rpm/min with no reverse. The maximum speed was set to 50 rpm and the minimum speed was 4 rpm. To test motor function in SOD1^G93A^ mice, acceleration was set to 5.0 rpm/min with no reverse. The maximum speed was set to 80 rpm and the minimum speed was 2 rpm. Each mouse was tested three times with at least 5-min breaks between trials. When comparing the ability of mice to stay on the rotarod, we only used the maximum time they spent on the rotating platform for each trial.

At least eight animals were tested on both the rotarod and hanging tests. To compare motor function between young adult mice, 5-month-old male wild-type and VAChT^Hyp^ mice were used. To compare motor function between middle-aged mice, 13-month-old female wild-type mice and VAChT^Hyp^ mice were examined. We examined male mice expressing SOD1^G93A^.

### Analysis of survival rates

All mice affected with ALS were regularly observed. Mice were euthanized when they were unable to right themselves back up after lying on their sides. A Kaplan–Meier log rank test was used to compare the lifespan between the groups of mice affected with ALS.

### Statistical analysis

Student’s *t* test and a one-way ANOVA followed by Tukey–Kramer and Kolmogorov-Smirnov tests were used to compare differences between groups. Data were expressed as the mean ± SE (standard error). *P* < 0.05 was considered statistically significant.

## Results

### VAChT is increased at NMJs of VAChT^Hyp^ mice

**Fig. 1:**
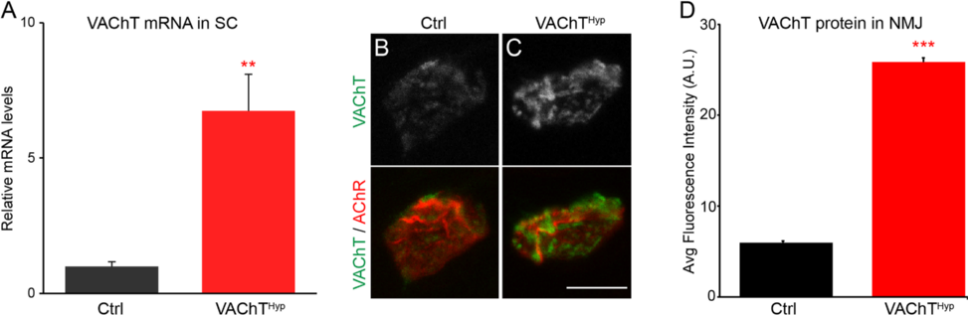
VAChT transcripts are increased in the spinal cord of VAChT^hyp^ compared to control mice (**a**). VAChT protein is also increased in motor axon nerve endings innervating skeletal muscles in VAChT^Hyp^ compared to control mice (**b**–**d**). Only male mice on a C57BL/6 background were used in these experiments. For qPCR, animals were 83 days old (*n* = 4 for control; *n* = 4 VAChT^hyp^). For NMJ analysis, animals were 9 days old (*n* = 4 for control; *n* = 4 VAChT^hyp^). *Scale bar* = 10 μm. *Error bar* = Standard Error. **P value <0.01

To assess the impact of altering acetylcholine (ACh) levels on the neuromuscular system, we examined a transgenic mouse line with increased expression of the vesicular acetylcholine transporter (VAChT) gene (*ChAT–ChR2–EYFP* [9, 21]; herein called VAChT^Hyp^). These mice have been shown to release threefold more synaptic ACh in the brain [9]. In this study, we only examined VAChT^Hyp^ heterozygous mice. We first confirmed that VAChT is expressed at higher levels in the spinal cord of VAChT^Hyp^ heterozygous mice using quantitative PCR (qPCR). As expected, we found VAChT messenger RNA (mRNA) significantly increased in the spinal cord of VAChT^Hyp^ mice compared to litter mate controls (Fig. 1a). We next used immunohistochemistry to examine the concentration of VAChT protein at neuromuscular junctions (NMJs) in the extensor digitorum longus (EDL) muscle. NMJs were visualized using fluorescently tagged α-bungarotoxin (fBTX), which binds selectively to nicotinic acetylcholine receptors (AChRs) located on the postsynaptic region of the NMJ. Using the same scanning parameters, VAChT immunofluorescence was invariably more intense at NMJs of VAChT^Hyp^ mice (Fig. 1c-d) compared to NMJs of control mice (Fig. 1b, d).

### Miniature endplate potential amplitudes are increased in VAChT^Hyp^ muscles

**Fig. 2:**
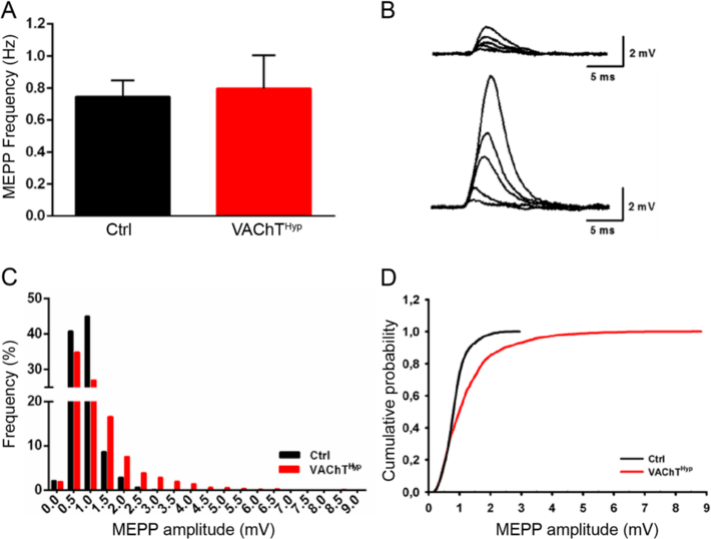
The impact of increasing ACh was determined by recording miniature endplate potentials (MEPPs) in the diaphragm of young adult control and VAChT^Hyp^ mice. The frequency of MEPPs is unchanged in VAChT^Hyp^ mice (**a**), with *error bar* representing the standard error. However, the average MEPP amplitude is higher in VAChT^Hyp^ (*n =* 2297 events) compared to that in control mice (*n =* 2444 events) (**b**–**d**). The amplitude of MEPPs was on average significantly higher in VAChT^Hyp^ mice using a Kolmogorov-Smirnov test (**d**; *P* value <0.001). Results are from 50 muscle fibers from five mice per genotype. *Error bar* = Standard Error. Four-month-old male mice on a C57BL/6 background were used for these experiments

To determine the impact of overexpressing VAChT on quantal size, we measured miniature endplate potentials (MEPPs) in the diaphragm muscle from 4-month-old controls and from VAChT^Hyp^ mice. While the frequency of MEPPs was unchanged (Fig. 2a), the amplitude of MEPPs was significantly higher in VAChT^Hyp^ compared to control mice (Fig. 2b, c). The mean MEPP amplitude was 0.86 ± 0.12 (mean ± SEM per muscle fibers per animal) mV in control mice (median = 0.75 mV) and 1.235 ± 0.18 mV in VAChT^Hyp^ mice (median = 1.244 mV). We also found that the frequency of large mini amplitudes increased in VAChT^Hyp^ mice (Fig. 2c). Reinforcing the notion that MEPP amplitudes are larger in VAChT^Hyp^ animals compared to controls, the cumulative probability plot (Fig. 2d) shows a shift to the right in the VAChT^Hyp^ curve (*P* < 0.001, Kolmogorov-Smirnov). These findings demonstrate that VAChT overexpression augments the amount of ACh loaded into and released from synaptic vesicles at NMJs.

### Normal development of NMJs in VAChT^Hyp^ mice

**Fig. 3:**
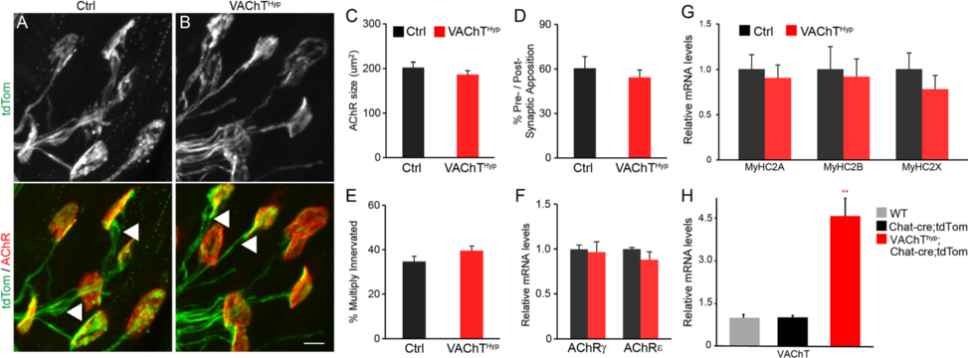
Developing NMJs are not affected in VAChT^Hyp^ mice. Multiple axons (*arrowheads*) innervating the same postsynaptic site in P9 EDL muscles from control and VAChT^Hyp^ mice (**a**, **b**). Comparison of structural features revealed no discernable differences between genotypes. The size of AChR clusters (**c**), apposition between the presynaptic (*green* in **a** and **b**) and postsynaptic apparatus (*red* in **a** and **b**), **d** and the incidence of multiply innervating NMJs (**e**) were unchanged in VAChT^Hyp^ mice. Transcripts for the gamma and epsilon subunits of AChRs were also unchanged in developing (P9) EDL and TA muscles from VAChT^Hyp^ mice (**f**). Similar levels of myosin heavy chains type 2A, 2B, and 2X were also found in TA + EDL of developing (P9) control and VAChT^Hyp^ mice (**g**). While VAChT mRNA is elevated in the spinal cord of VAChT^hyp^; Chat-cre;tdTom mice, it is unchanged in the spinal cord of 3-month-old Chat-cre;tdTom mice compared to wild-type mice (**h**). All mice used in this study were maintained on a C57BL/6 background. The following mice were used for NMJ analysis: Ctrl = Chat-cre;tdTom and VAChT^Hyp^ = VAChT^hyp^;Chat-cre;tdTom. At least four mice were used for each genotype and per experiment. At least 25 NMJs per animal were examined. *Scale bar* = 10 μm. *Error bar* = Standard Error. *P* = 0.008

Several lines of evidence indicate that ACh acts as an anti-synaptogenic factor at NMJs [4, 5, 7]. We thus asked if increased ACh levels affect developing NMJs in the predominantly fast-twitch EDL muscle. To facilitate analysis of motor nerve endings, we generated VAChT^Hyp^ mice expressing tdTomato specifically in cholinergic neurons. This was accomplished by mating VAChT^Hyp^ mice with ChAT-IRES-Cre [17] and tdTomato [18] mice. Control and VAChT^Hyp^ mice are referred herein as ChAT-Cre;tdTomato and VAChT^Hyp^;ChAT-Cre;tdTomato, respectively. To ascertain that VAChT expression is not affected by expression of Cre from the ChAT (Chat-Cre) locus and tdTomato from the Rosa locus (tdTomato), we examined VAChT mRNA levels in the spinal cord of wild-type, Chat-Cre;tdTomato, and VAChT^Hyp^;Chat-Cre;tdTomato mice. While VAChT transcripts were significantly elevated in the spinal cord of VAChT^Hyp^;Chat-Cre;tdTomato mice, there was no difference between wild-type and Chat-Cre;tdTomato mice (Fig. 3h). We thus used VAChT^Hyp^ and control mice expressing tdTomato to examine NMJs at postnatal day 9 (P9). At this age, NMJs are still in the process of developing from a small plaque, often occupied by multiple innervating α-motor nerve endings, to a large pretzel-like structure [22]. We found that increased VAChT expression had no effect on the development of NMJs. The size of AChR clusters, the percent apposition between pre- and postsynaptic sites, and the incidence of multiply innervated NMJs are similar in the EDL muscle of VAChT^Hyp^ and control mice (Fig. 3a–e). Next, we asked if increased ACh affects expression of the gamma and epsilon subunits of AChRs in developing EDL muscles. The epsilon subunit replaces the gamma subunit as NMJs mature [23], altering the functional properties of AChRs [24, 25], and thus serves as a functional marker for maturing NMJs. Our analysis revealed similar levels of both the gamma and epsilon subunits in VAChT^Hyp^ mice compared to control mice of the same age (Fig. 3f). Collectively, these findings demonstrate that the structural and functional maturation of the NMJ are unaffected by higher levels of synaptic ACh.

Nerve activity has been well documented to influence the maturation and specification of muscle fiber types [26]. There are four major types of skeletal muscle fibers that can be identified based on their expression of myosin heavy chain (MyHC) isoforms (type 1, 2A, 2X, or 2B) [27]. We thus asked if altering levels of ACh affect muscle biogenesis, independently of changes at NMJs. In the tibialis anterior (TA) and EDL muscles of 9-day-old VAChT^Hyp^ mice, mRNA levels for MyHC type 2A, 2B, and 2X were similar to those in control mice (Fig. 3g). These findings show that the increased level of synaptic ACh in VAChT^Hyp^ does not alter the normal development of muscle fibers and NMJs in the TA and EDL muscles.

### NMJs exhibit age-related changes in young adult VAChT^Hyp^ mice

**Fig. 4:**
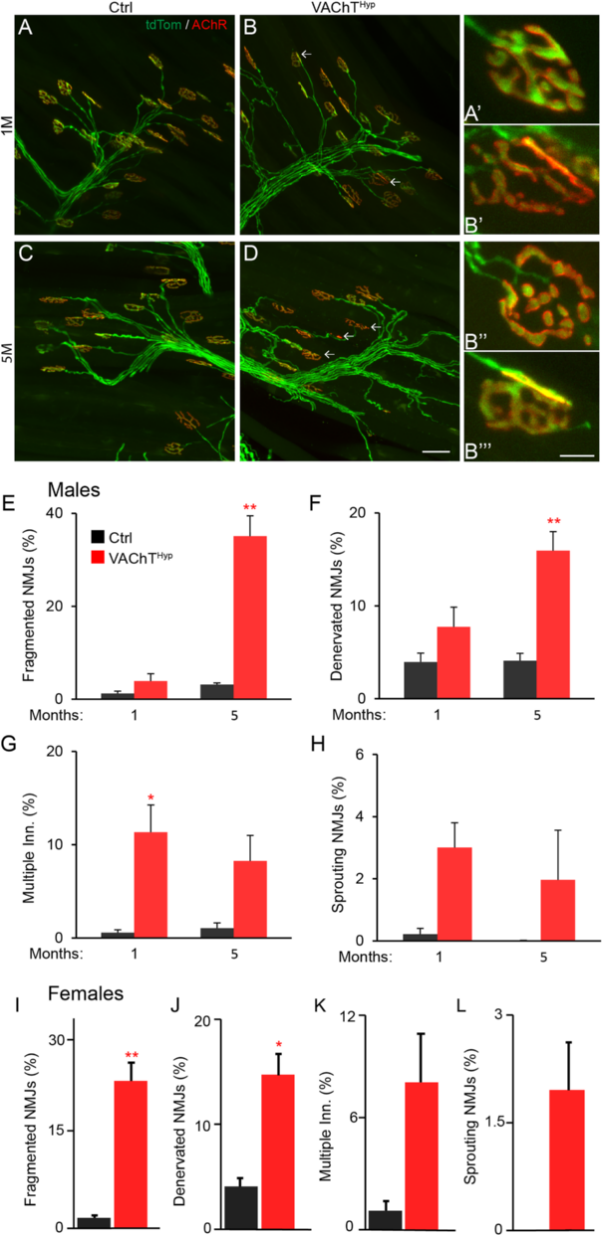
NMJs progressively degenerate in adult VAChT^Hyp^ mice. At 1 month of age, there is an increased incidence of NMJs with age- and disease-related structural features in the EDL muscle of VAChT^Hyp^ compared to control mice (**a**, **b**, **e**–**h**). By 5 months of age, there is an additional increase in the number of fragmented and denervated NMJs in male (**e**–**h**) and female (**i**–**l**) VAChT^Hyp^ mice. α-motor axons expressed tdTomato in these mice (*green* in **a**–**d**). *Scale bar* = 50 μm (**a**–**d**), 10 μm (**a’**–**b”’**). All mice used in this study were maintained on a C57BL/6 background and only male mice were examined. Ctrl = Chat-cre;tdTom and VAChT^Hyp^ = VAChT^hyp^;Chat-cre;tdTom. At least four mice were used for each genotype and per experiment. *Error bar* = Standard Error. *P value <0.05, **P value <0.01

**Fig. 5:**
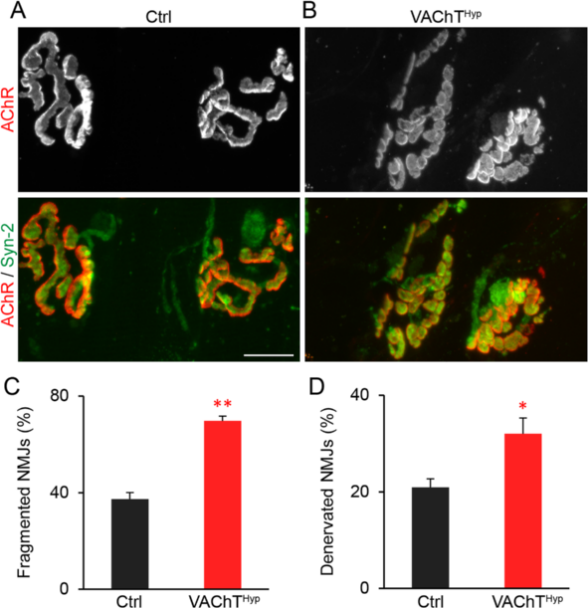
Accelerated aging of NMJs in aging VAChT^Hyp^ mice. α-motor nerve endings (*green* in **a** and **b**) were visualized using an antibody against synaptotagmin-2 (Syn-2; *green*). There were more fragmented and denervated NMJs found in 19-month-old VAChT^Hyp^ (**b**–**d**) compared to control (**a**, **c**–**d**) mice of the same age and sex. Four mice per genotype were examined and at least 50 NMJs per animal analyzed. *Scale bar* = 20 μm. *Error bar* = Standard Error. *P value <0.05, **P value <0.01

In adulthood, the NMJ undergoes functional and structural changes due to diseases, injury, aging, and exercise [19, 28]. Since all these different conditions inevitably affect cholinergic transmission, we assessed the impact of moderately increasing synaptic ACh on adult NMJs. In the EDL muscle of 1-, 5-, and 19-month-old VAChT^Hyp^ and aged-matched control mice, we examined NMJs for fragmentation, denervation, sprouting of motor axon nerve endings, and innervation by multiple motor axons. These cellular characteristics are a hallmark of NMJs in aged EDL muscles. We thus refer to them as age-related structural features. In 1-month-old male ﻿VAChT^Hyp^ mice, ﻿﻿there was no change in the number of fragmented and denervated NMJs compared to control mice of the same age and sex (Fig. 4a, b, e, f). However, NMJs were often found multiply innervated (Fig. 4g) and with motor axons sprounting beyond the postsynaptic site (Fig. 4h) in 1-month-old VAChT^Hyp^ mice.  By 5 months of age, fragmented and denervated NMJs were more prevalent in male VAChT^Hyp^ mice (Fig. 4d–f) compared to age- and sex-matched control and 1-month-old VAChT^Hyp^ mice (Fig. 4c, e, f). The number of multiply innervated NMJs and axons sprouting beyond the postsynaptic site was similar between 1- and 5-month-old VAChT^Hyp^ mice (Fig. 4g, h). NMJs were similarly affected in 5-month-old female VAChT^Hyp^ mice (Fig 4i–l). At 19 months of age, the incidence of NMJs with deleterious age-related structural changes further increased in female VAChT^Hyp^ mice compared to control mice (Fig. 5a–d). Contrary to our expectations, these findings show that moderately increasing synaptic ACh accelerates aging of NMJs.

**Fig. 6:**
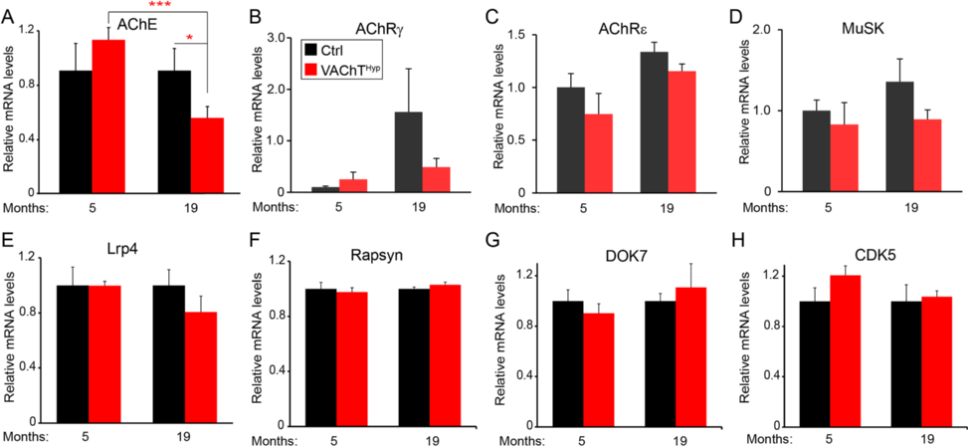
Analysis of NMJ-associated genes in the EDL muscle of 5- and 19-month-old control and VAChT^hyp^ mice. This analysis showed that only AChE is altered and specifically in 19-month-old VAChT^hyp^ mice (**a**). Transcripts for AChRγ, AChRε, MuSK, Lrp4, rapsyn, DOK7, and CDK5 were expressed at similar levels in the EDL muscle of VAChT^Hyp^ compared to control mice at 5 and 19 months of age (**b–h**). At least four female mice per genotype maintained on a C57BL/6 background were used to compare transcript levels. *Error bar* = Standard Error. *P = 0.038, ***P = 0.00063

We next asked if structural alterations at NMJs in VAChT^Hyp^ mice are the result of changes in key molecules with critical functions at NMJs. For this, we examined mRNA levels for eight NMJ-associated molecules in the EDL muscle of 5- and 19-month-old mice. Although acetylcholinesterase (AChE) was expressed at similar levels in the EDL muscle of 5-month-old control and VAChT^Hyp^ mice, AChE was significantly reduced in aged VAChT^Hyp^ (Fig. 6a). In contrast, mRNA for the gamma and epsilon nicotinic AChR subunits (Fig. 6b, c) [29], the muscle-specific kinase (MuSK) (Fig. 6d) [30], the LDL receptor-related protein 4 (LRP4) (Fig. 6e), rapsyn (Fig. 6f), the docking protein 7 (Dok7) (Fig. 6g), and the cyclin-dependent kinase 5 (CDK5) (Fig. 6h) were unchanged in the EDL muscle of 5- and 19-month-old VAChT^Hyp^ mice compared to control mice of the same age.

### NMJ degeneration precedes motor deficits in VAChT^Hyp^ mice

**Fig. 7:**
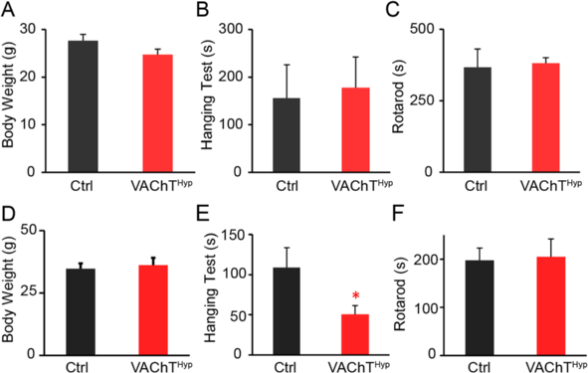
VAChT^Hyp^ mice exhibit motor deficits in middle age. At 5 months of age, VAChT^Hyp^ mice do not exhibit body weight loss (**a**) or motor function deficits (**b**, **c**). Thirteen-month-old VAChT^Hyp^ mice weigh the same (**d**) and perform equally well on a rotarod (**f**) as control mice of the same age. However, the hanging test revealed motor deficits in 13-month-old VAChT^Hyp^ mice (**e**). Only female mice maintained on a C57BL/6 background were used for these experiments. Eight control and 12 VAChT^hyp^ mice were examined on the hanging and rotarod tests at both ages. *Error bar* = Standard Error. *P value <0.05, **P value <0.01

The accelerated aging of NMJs suggests that motor function may be compromised in VAChT^Hyp^ mice. To explore this possibility, we tested motor function of 5- and 13-month-old VAChT^Hyp^ and control mice by measuring the latency to fall from a wire mesh and from a rotating platform. At both ages, we found no difference in body weight between genotypes (Fig. 7a, d). At 5 months of age, VAChT^Hyp^ and control mice performed equally well on both tests (Fig. 7b, c). However, 13-month-old VAChT^Hyp^ mice were unable to support their own weight on an inverted wire mesh as long as age- and sex-matched control mice (Fig. 7e). However, 13-month-old VAChT^Hyp^ mice spent the same amount of time on the rotarod as control mice of the same age (Fig. 7f). These findings indicate that, in addition to accelerating degeneration of NMJs, increasing synaptic ACh compromises motor function.

### NMJ degeneration precedes muscle atrophy in VAChT^Hyp^ mice

**Fig. 8:**
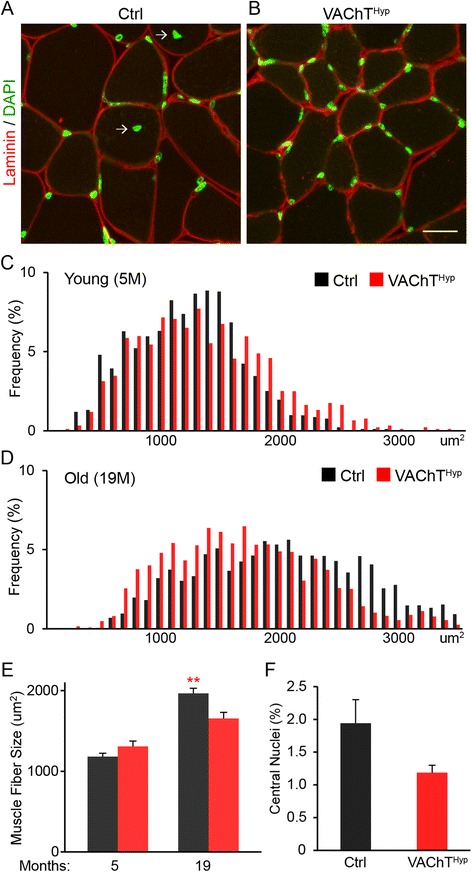
Analysis of muscle fibers in young adult and old VAChT^Hyp^ mice. Muscle fibers were examined in VAChT^Hyp^ and control mice. Muscle fibers in the TA muscle were stained with an antibody against laminin to visualize their size (*red*) and labeled with the nuclei marker, DAPI (*green*), to determine if muscle fibers had undergone bouts of degeneration and regeneration (**a**, **b**). Images are from 19-month-old mice. The size of muscle fibers is similar in 5-month-old control and VAChT^Hyp^ mice (**c**, **e**). However, muscle fibers are smaller in 19-month-old VAChT^Hyp^ mice (**d**, **e**). There are also no significant differences in the number of muscle fibers with centrally located myonuclei between 19-month-old VAChT^Hyp^ control mice (**f**). At least four female mice per genotype maintained on a C57BL/6 background were used to compare transcript levels. *Scale bar* = 20 μm. *Error bar* = Standard Error. *P value <0.05

**Fig. 9:**
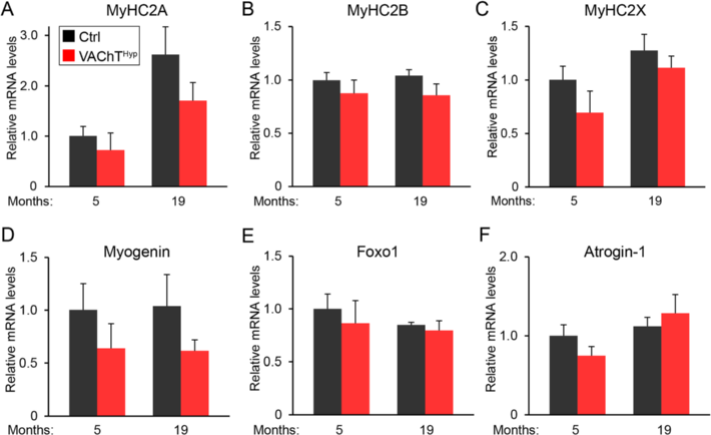
Analysis of transcripts associated with muscle fiber identity (MyHC2A, MyHC2B, MyHC2B) and markers of muscle regeneration and atrophy (Myogenin, Foxo1, Atrogin-1) revealed no change in the EDL muscle of VAChT^Hyp^ compared to control mice at 5 and 19 months of age (**a**–**f**). At least four female mice per genotype maintained on a C57BL/6 background were used to compare transcript levels. *Error bar* = Standard Error

We next examined the effect of increasing cholinergic activity on muscle fibers’ structure and molecular composition. To start, we stained 14 μm muscle cross sections from the TA muscle of 5- and 19-month-old mice with an antibody against laminin to visualize and measure the perimeter of all muscle fibers. This analysis showed that the size of muscle fibers is unchanged in 5-month-old VAChT^Hyp^ mice compared to control mice of the same age (Fig. 8c, e). However, muscle fibers were slightly smaller in 19-month-old VAChT^Hyp^ mice (Fig. 8a, b, d, e). To determine if the reduced size of muscle fibers in 19-month-old VAChT^Hyp^ mice is due to atrophy, we examined the location of myonuclei using 4′,6-diamidino-2-phenylindole (DAPI). Myonuclei located away from the peripheral membrane mark degenerating and regenerating muscle fibers. In 5-month-old VAChT^Hyp^ and control mice, all myonuclei were found adjacent to the peripheral membrane. While a small number of muscle fibers contained myonuclei near the middle of the cytoplasm in 19-month-old mice, there was no difference between VAChT^Hyp^ and control mice (Fig. 8f). To further assess the effect of increasing synaptic ACh on muscle fibers, we examined levels of MyHC types 2A, 2B, and 2X in the EDL muscle. Transcripts for these genes were similarly expressed in 5- and 19-month-old VAChT^Hyp^ and control mice (Fig. 9a–c). Moreover, levels of myogenin, forkhead box protein O1 (Foxo1), and Atrogin1 [31–33], markers of atrophying and regenerating muscle fibers, were also unchanged in the EDL muscle of VAChT^Hyp^ compared to control mice of the same age (Fig. 9d–f). These findings indicate that the deleterious age-related structural features found at NMJs do not result in significant myogenic changes in VAChT^Hyp^ mice.

### Presynaptic degeneration occurs in the absence of obvious changes in motor neurons in VAChT^Hyp^ mice

**Fig. 10:**
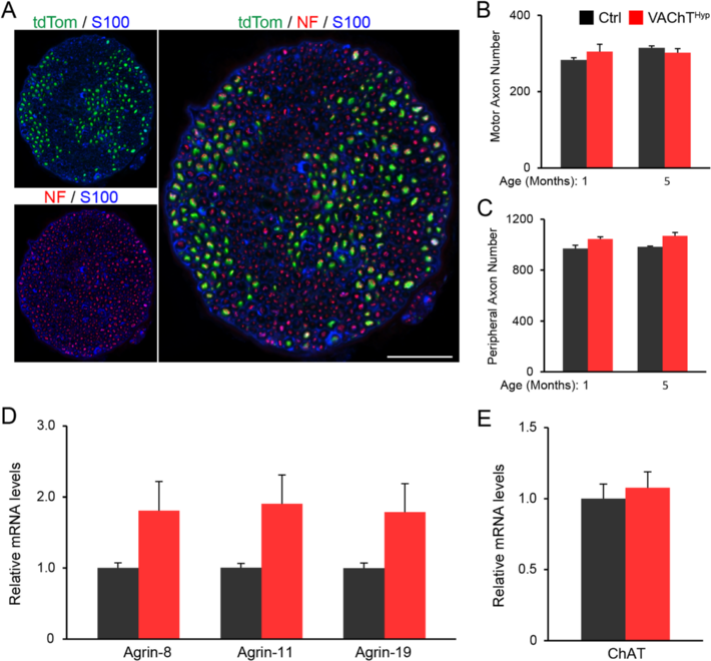
The number of motor axons and expression of molecules with critical function at the NMJ are unchanged in VAChT^Hyp^ mice. Cross sections (10 μm) of the common peroneal nerve expressing tdTomato in motor axons were also labeled with antibodies against neurofilament (NF) and the Schwann cell marker, S100, (**a**). There was no difference found in the number of motor axons (**b**) or peripheral axons (**c**) between control and VAChT^Hyp^ mice at 1 and 5 months of age. The expression of neuronal-agrin isoforms (**d**) and ChAT (**e**) are not significantly altered in the spinal cords of 3-month-old VAChT^Hyp^ mice. At least four male mice maintained on a C57BL/6 background were used per genotype for axonal counts and expression analysis. *Scale bar* = 50 μm. *Error bar* = Standard Error

We next asked if degeneration of NMJs results in loss of motor neurons. For this purpose, we compared the number of motor axons present in the peroneal nerve of control and VAChT^Hyp^ mice expressing tdTomato [18] specifically in cholinergic neurons [17]. To count motor axons, we generated 10-μm cross sections of the common peroneal nerve, a mixed nerve that innervates the TA, EDL, and other hind limb muscles (Fig. 10a). This analysis revealed no difference in the number of motor axons in 1- and 5-month-old VAChT^Hyp^ mice compared to control mice of the same age (Fig. 10b). The total number of peripheral axons, including sensory afferents, was also unchanged in VAChT^Hyp^ mice (Fig. 10c).

As a response to presynaptic degeneration and increased expression of VAChT, motor neurons may increase expression of agrin isoforms that function to stabilize the NMJ [34]. To explore this possibility, we examined transcripts for neural agrin isoforms (z-agrin) in the spinal cord of 5-month-old VAChT^hyp^ and control mice. While we found that z-agrin isoforms were not statistically altered in the spinal cord of VAChT^Hyp^ mice compared to control mice, it is worth noting that all three z-agrin isoforms were moderately and consistently higher, approximately 1.8-fold for all isoforms in VAChT^Hyp^ mice (Fig. 10d). If motor neurons were to increase these z-agrin isoforms in VAChT^Hyp^ mice, it would likely be to stabilize nAChRs on the postsynaptic region, and thus prevent further fragmentation and denervation of NMJs. It is also plausible that overexpression of VAChT results in induction of genes involved in the biosynthesis of ACh. This seems unlikely, however, since the choline acetylcholine transferase (ChAT) is expressed at similar levels in the spinal cord of VAChT^Hyp^ and control mice (Fig. 10e). Together with the lack of axonal degeneration, these findings indicate that increasing synaptic ACh directly affects the structural integrity of the NMJ.

### Increased cholinergic transmission accelerates ALS pathogenesis

**Fig. 11:**
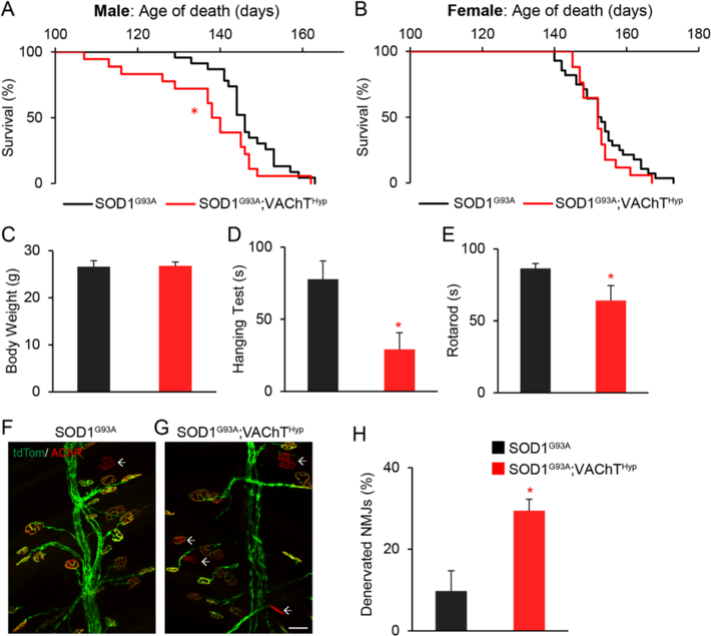
Overexpression of VAChT accelerates NMJ degeneration and death of male mice expressing SOD1^G93A^. The lifespan of male and female SOD1^G93A^ alone and crossed with VAChT^Hyp^ mice was determined. Overexpression of VAChT selectively accelerated death of male SOD1^G93A^ mice (**a**) without altering their weight (**c**). The average lifespan of female mice was unchanged (**b**). Male SOD1^G93A^;VAChT^Hyp^ mice also exhibited accelerated motor deficits at 75 days of age (**d**, **e**) and accelerated NMJ degeneration at 90 days of age (**f**–**h**). All mice were maintained on a C57BL/6 background. For survival and motor tests, SOD1^G93A^ and SOD1^G93A^;VAChT^Hyp^ mice without fluorescence protein were used. Survival curves are based on as follows: Males = 31 SOD1^G93A^ and 21 SOD1^G93A^;VAChT^Hyp^. Females = 41 SOD1^G93A^ and 20 SOD1^G93A^;VAChT^Hyp^. Motor tests were performed on 10 male SOD1^G93A^ and 10 male SOD1^G93A^;VAChT^Hyp^ mice. NMJ analysis was performed on four male mice per genotype expressing tdTomato in motor neurons. At least 50 NMJs were examined in the EDL muscle per animal. *Scale bar* = 50 μm. *Error bar* = Standard Error. *P value <0.05

Early and progressive degeneration of NMJs is a hallmark of amyotrophic lateral sclerosis (ALS) [35]. In addition, dysregulated cholinergic transmission has become recognized as a factor that exacerbates ALS-related pathologies in mice and in cultured motor neurons [36–39]. To determine the impact of altering cholinergic transmission on ALS-related pathological features, we crossed VAChT^Hyp^ mice with SOD1^G93A^ transgenic mice (SOD1^G93A^;VAChT^Hyp^). Increasing cholinergic transmission selectively accelerated death of male SOD1^G93A^ mice by approximately 10 days (Fig. 11a). Although male SOD1^G93A^;VAChT^Hyp^ mice weighed the same as SOD1^G93A^ mice (Fig. 11c), they had diminished motor function before the onset of neurological symptoms (Fig. 11d, e). At 75 days of age, male SOD1^G93A^;VAChT^Hyp^ mice were unable to stay as long as the SOD1^G93A^ mice on an inverted wire mesh, a test that measures motor function (Fig. 11d, e). Along with early motor deficits, we found that motor axons prematurely vacate NMJs in 90-day-old male SOD1^G93A^;VAChT^Hyp^ (Fig. 11f–h). These early behavioral and cellular changes were specific to male mice; we found no differences in survival between SOD1^G93A^;VAChT^Hyp^ and SOD1^G93A^ female mice (Fig. 11b). We also found no changes in copy numbers between SOD1^G93A^ and SOD1^G93A^;VAChT^Hyp^ mice, strongly suggesting that increased levels of ACh accelerate degeneration of the neuromuscular system in male SOD1^G93A^ mice.

## Discussion

### Impact of increasing synaptic ACh on NMJs, muscle fibers, and motor neurons

VAChT plays a critical role in the storage of ACh in synaptic vesicles [10, 40, 41]. In VAChT^Hyp^ mice, VAChT and ACh secretion is increased in brain tissue [9]. Our qPCR and immunofluorescence analysis revealed that VAChT is similarly increased in the spinal cord and at the NMJ. Previous experiments have examined the amount of ACh released from hippocampal slices from these mice [9]. However, these studies did not address whether increased expression of VAChT results in more ACh loaded into synaptic vesicles in vivo and in motor neurons. Our finding that MEPP amplitudes are increased in VAChT^Hyp^ mice strongly suggests that overexpressing VAChT increases ACh accumulation in matured synaptic vesicles. These results suggest that at least a population of mature synaptic vesicles can accumulate higher amounts of ACh upon increased levels of transporter protein. Vesicular ACh accumulation by VAChT is complex and thought to be tightly regulated [42]. Our data suggest that potential changes in expression or trafficking of VAChT to synaptic vesicles can regulate the amount of ACh secreted by nerve endings by direct regulation of neurotransmitter accumulation in synaptic vesicles. Previous reports have shown that overexpression of VAChT in immature nerve-muscle Xenopus cultures increases both amplitude and frequency of MEPPs. This was interpreted as incorporation of VAChT in immature synaptic vesicles can both increase the amount of ACh stored in vesicles as well as increase the number of filled vesicles available for spontaneous release [43]. We did not observe an increased frequency of MEPPs in adult mice overexpressing VAChT, suggesting that the population of vesicles available for spontaneous release is unchanged in the NMJ.

In addition to its function in neurotransmission, ACh plays a critical role in the development of the NMJ. ACh acts as an anti-synaptogenic molecule by decreasing the stability of AChRs, and through this activity, it is believed to function together with the neural-derived factor, z-agrin, in sculpting the NMJ [5]. In this regard, while deletion of genes that prevent the synthesis [5, 7] and transport [41] of ACh into synaptic vesicles cause significant changes at NMJs, the impact of increasing synaptic ACh levels on developing and adult NMJs had not been examined. We now show that the rate of synapse elimination, the size of AChR clusters, and their apposition with presynaptic sites are unchanged in developing VAChT^Hyp^ mice. In addition, we found no difference in the development of muscle fibers. The normal development of NMJs and muscle fibers in VAChT^Hyp^ mice may be attributed to different factors. The additional ACh released by motor neurons in VAChT^Hyp^ mice may not change muscle action potentials as ACh is already in excess during development [24]. Another plausible explanation, though not necessarily mutually exclusive, is that synaptic ACh and z-agrin are present at saturating levels compared to nAChRs during development. Thus, developing NMJs and muscle fibers are likely immune to further increasing ACh at the synaptic cleft, as is the case in VAChT^Hyp^ mice. Regarding synaptic elimination, all motor neurons in VAChT^Hyp^ mice are likely to retain their “competitive vigor” [44, 45] since ACh is likely increased by the same magnitude in all NMJs. Hence, the time motor axons spend competing for the same target would not be expected to change in VAChT^Hyp^ compared to those in control mice.

In adulthood, the incidence of NMJs with deleterious structural features increases with aging in control mice, and those features are more pronounced in VAChT^Hyp^ mice. These findings indicate that NMJs within the EDL muscle vary in their susceptibility to increased levels of ACh at the synaptic cleft. The EDL muscle is primarily composed of muscle fibers that express myosin heavy chain type 2A and type 2B [28]. Thus, it is plausible that one of these muscle fiber type is more susceptible to increased synaptic ACh levels, a possibility not addressed in this work. Another possibility is that NMJs receiving less resource within a motor unit fail to adequately repair following structural changes driven by increased ACh at their synaptic clefts. In this regard, it has been shown that NMJs within the same motor unit respond differently to aging [28]. It is also possible that some motor neurons are more active, resulting in earlier degeneration of NMJs that make up their motor unit. Thus, an increase in the amount of synaptic ACh together with less functional z-agrin in adult NMJs would result in structural changes due to increased destabilization of AChRs. However, this conclusion is not supported by the lack of changes in transcript for the gamma AChR subunit, which is closely associated with increased internalization and degradation of AChRs. While NMJs are clearly affected in adult VAChT^Hyp^ mice, it remains possible that cholinergic dysregulation elsewhere in the peripheral and central nervous system, including the spinal cord, may cause or contribute to the precocious degeneration of NMJs observed in young adult VAChT^Hyp^ mice. Regardless, the results in this paper do not support previous suggestions [1, 15] that decreased neuromuscular function as a result of aging and ALS should be reversed by increased release of ACh at the NMJ synaptic cleft. Instead, these findings raise the possibility that the age-related increase in cholinergic transmission reported by several published studies contribute to the degeneration of NMJs [46–50].

It is interesting that old VAChT^Hyp^ mice present with worse muscle function than age-matched control mice. This is in striking contrast with better performance of young VAChT^Hyp^ mice on the treadmill [9]. Interestingly, recent analysis of the cardiac muscle in VAChT^Hyp^ mice showed that they have preserved cardiac function when challenged with angiotensin II, a model of heart failure [51]. These data suggest that the better performance of VAChT^Hyp^ mice on the treadmill may be a result of better cardiovascular fitness.

### Male SOD1^G93A^ mice are selectively susceptible to increased ACh levels

We also showed that increasing cholinergic transmission selectively affects male mice harboring SOD1^G93A^. Increased synaptic ACh causes NMJs to prematurely degenerate and accelerates death of male SOD1^G93A^ mice. While our findings demonstrate that increasing synaptic ACh does not affect female SOD1^G93A^ mice, it is worth noting that SOD1^G93A^ mice acquire neurological symptoms by 90 days and die within the first 160 days of life. Hence, it remains possible that increasing ACh at NMJs affects the initiation and progression of ALS-related pathology in long-lived female mice expressing mutant genes known to cause ALS, in addition to further exacerbating neurological symptoms in long-lived male mice expressing the same mutant genes. Irrespective, our data corroborates published findings indicating that male animals and humans are more susceptible to ALS [52, 53], and factors that suspect of altering the initiation and progression of the disease [54–58].

### Significance to understanding aging- and disease-related changes at NMJs

It is now recognized that cholinergic dysfunction occurs in neurons harboring ALS-causing mutant genes and during normal aging [1, 35, 36, 59]. In addition, perisynaptic Schwann cells were recently shown to be uniquely sensitive to altered cholinergic transmission prior to the initiation of ALS-related symptoms in a mouse model for the disease [37]. The identification of mutations in cholinergic receptors in patients with ALS provides additional evidence that changes in cholinergic transmission directly drives degeneration of motor neurons and their NMJs [38, 39, 60]. In this regard, the only FDA-approved drug to treat ALS, riluzole, was recently shown to bind and to block the activity of muscle nicotinic AChRs obtained from patients with the disease [61]. The results presented in this paper provide evidence that increased cholinergic tone deteriorates NMJs in a mouse model of ALS and during normal aging. Thus, modulation of cholinergic transmission should be considered in approaches aimed at preventing degeneration of the motor system during the progression of ALS and normal aging.

## Conclusions

This study sought to determine the impact of increasing synaptic ACh levels on developing, aging, and ALS-afflicted NMJs using mice expressing multiple copies of VAChT. We showed that while NMJs develop normally in VAChT^Hyp^ mice, they progressively degenerate as the mice aged. This study also revealed that destruction of NMJs precedes degeneration of muscle fibers and motor neurons during aging and in mice harboring an ALS-causing mutant gene. Together, the data in this paper demonstrate that dysregulated levels of ACh cause pathophysiological changes at NMJs and contribute to loss of motor function during aging and the progression of ALS.

## Abbreviations

ALS: Amyotrophic Lateral Sclerosis
CHAT: Choline Acetyltransferase
CRE: Causes Recombination
MEPP: Miniature Endplate Potential
NMJ: Neuromuscular Junction
SOD1^G93A^: Superoxide Dismutase 1 mutated from a glycine to alanine at codon 93
VAChT: Vesicular Acetylcholine Transporter
VAChT^Hyp^: Heterozygous Chat-ChR2-EYFP
YFP: Yellow Fluorescence Protein
